# A Non-Secreting Pituitary Adenoma That Changed to a Prolactinoma

**DOI:** 10.3390/clinpract14040106

**Published:** 2024-07-04

**Authors:** Cristina Santiago-Vazquez, Nuria Palacios-Paino, Fernando Cordido

**Affiliations:** 1 Servicio Endocrinología y Nutrición, Hospital Universitario A Coruña, 15006 A Coruña, Spain; cristina.santiago.vazquez@sergas.es (C.S.-V.); nuria.palacios.paino@sergas.es (N.P.-P.); 2 Grupo Fisiopatoloxía Endócrina, Nutricional e Médica (FENM), Facultad de Ciencias de la Salud, Universidade da Coruña, 15006 A Coruña, Spain; 3 Instituto de Investigación Biomedica (INIBIC), Centro de Investigaciones Científicas avanzadas (CICA), Universidade da Coruña, 15006 A Coruña, Spain

**Keywords:** pituitary adenomas, non-secreting pituitary adenoma, prolactinomas, macroprolactinomas

## Abstract

Pituitary adenomas (PAs) are the third most common brain tumors in adults right after meningiomas and gliomas. Taking into account their hormonal activity in vivo, they can be divided in functioning PAs, which secrete hormones, and nonfunctioning pituitary adenomas (NFPAs), which are not associated with increased hormone secretion. We present the case of a man diagnosed with pituitary apoplexy. A transsphenoidal surgery was performed with subtotal removal of the mass. Pituitary hormones were measured before and after the procedure on several occasions, showing always normal PRL values, so he was diagnosed with a clinically NFPA. Two years later, the patient noticed a visual deficit. A new magnetic resonance imaging study was performed, showing adenomatous recurrence, and the patient underwent a new surgery. After this, hormonal evaluation revealed high levels of PRL on several occasions. After treatment with cabergoline was started, PRL levels normalized, the visual deficit improved, and there was a slight adenoma reduction. This case report represents an exception to the paradigm that in the presence of a macroadenoma and normal PRL levels (avoiding the “hook effect”), a prolactinoma can be discarded. Moreover, it stresses the importance of comprehensive, regular, and lifelong surveillance of patients with NFPAs and the close monitoring of serum PRL.

## 1. Introduction

Pituitary adenomas (PAs) are the most common neoplasms of the sellar region, and taking into account their hormonal activity in vivo, they can be divided in functioning PAs, which secrete pituitary hormones autonomously, and nonfunctioning pituitary adenomas (NFPAs), which are not associated with increased hormone secretion [1]. PAs are present in approximately 10% of people in the general population on imaging studies or at autopsy. The great majority of these are microadenomas [2]. Clinically nonfunctioning PAs are present in approximately 1 in 1100 individuals in the general population. Of these, 48% are macroadenomas [3,4]. Macroadenomas can cause mass effect, consisting of visual field defects, headache, or hypopituitarism. People with hypopituitarism, including GH deficiency, due to PA have an approximately two-fold increase in mortality compared with the general population [5,6]. NFPAs account for approximately 30% of PAs that come to clinical attention and cause symptoms, and they arise from cells of gonadotroph lineage in about 80% of patients [7]. Prolactin-secreting PA (prolactinomas) account for approximately 53% of PAs and arise from cells of lactotroph lineage [8] Compared with men, women are approximately ten times more likely to develop prolactinomas and are diagnosed at a younger age; however, the tumors tend to be smaller [4]. Serum prolactin (PRL) should be measured to evaluate for prolactinoma [9]. These tumors secrete PRL in proportion to their size. A PA with a circulating PRL greater than 250 ng/mL is considered a prolactinoma. Other causes of an elevated serum PRL level, with values below 250 ng/mL, include drugs, pregnancy, kidney failure, liver failure, polycystic ovary syndrome, and primary hypothyroidism. Alternatively, PRL levels may be elevated due to a “stalk effect”. PRL secretion is normally inhibited by hypothalamic dopamine. Patients with an NFPA compressing the pituitary stalk may develop hyperprolactinemia due to disruption of dopamine outflow through the pituitary stalk (stalk effect) [9]. In patients with stalk effect, serum prolactin is generally below 150 ng/mL [9]. Artifactually low values of PRL occur when a very high serum PRL level saturates both the capture and signal antibodies used in immunoradiometric and chemiluminescent assays, preventing the binding of the two antibodies. This phenomenon is called the “hook effect”. Most authors agree that in the presence of a macroadenoma and a confirmed normal PRL level (after avoiding the “hook effect”), a prolactinoma can be discarded [4]. 

We present the clinical case of an initially non-secreting pituitary adenoma that changed to a prolactinoma, an entity that, as far as we know, has not been previously described in clinical practice.

## 2. Materials and Methods

The patient is a 53-year-old man whose most prominent personal background consisted in a cured hepatitis C virus infection and gastroesophageal reflux. He was initially evaluated in July 2018 in the Emergency Room for a headache that he reported to be more intense than usual, plus vomiting and fever of three days of evolution. He did not notice any visual deficit nor any other neurological symptom. At the physical examination, he presented a score of 15 on the Glasgow Coma Scale (GCS), cranial pairs examination was normal, and the perimetry test was also apparently normal.

**Figure 1:**
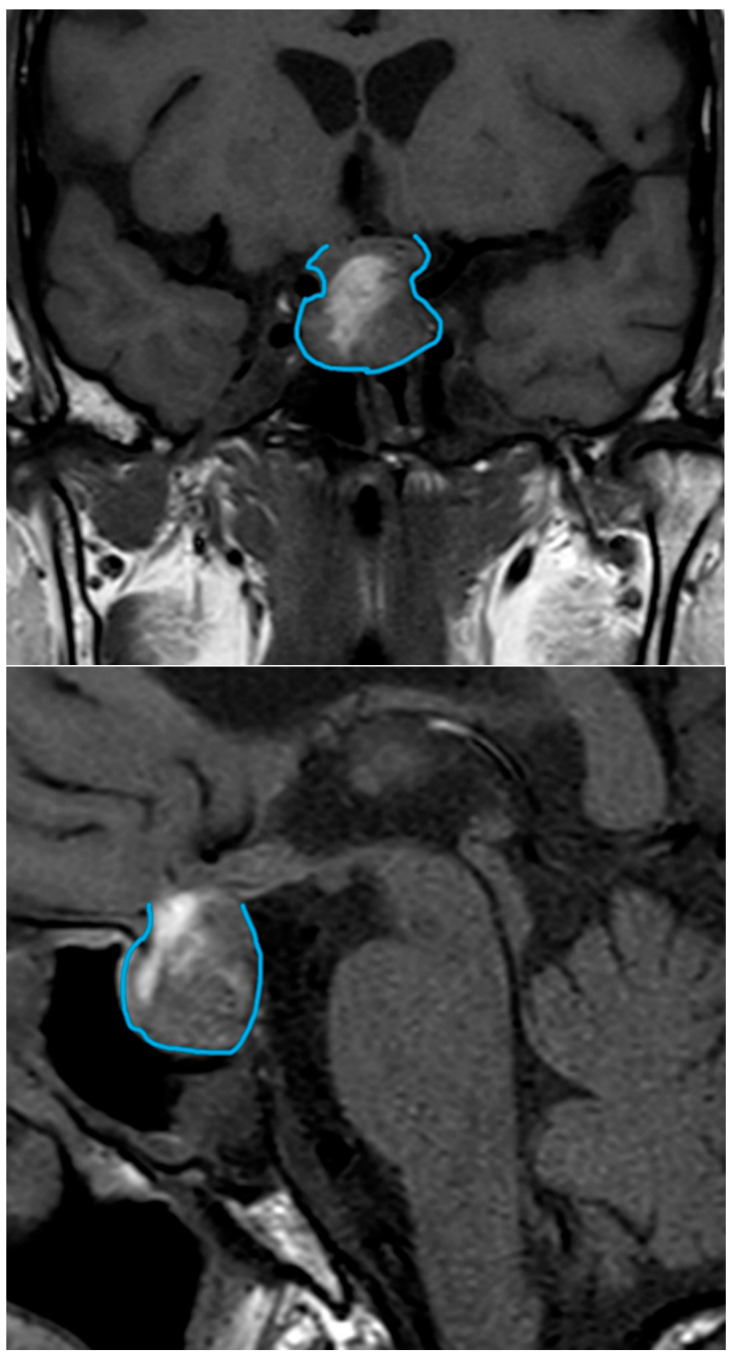
MRI, July 2018. Macroadenoma (20 × 20 × 18 mm) with signs of pituitary apoplexy and an invasion of not only the optic chiasm but also the hypothalamus and partially the left cavernous sinus. The tumor is delineated in all imaging studies. MRI, magnetic resonance imaging.

A computed tomography (CT) scan was performed, revealing a pituitary macroadenoma with cystic–hemorrhagic degeneration and compression of the optic chiasm. To complete the study, magnetic resonance imaging (MRI) was requested. MRI showed a macroadenoma (20 × 20 × 18 mm) with signs of pituitary apoplexy and an invasion of not only the optic chiasm but also the hypothalamus and partially the left cavernous sinus (Figure 1).

Furthermore, in laboratory studies, no hormone hypersecretion was found (T4, TSH, FSH, LH, PRL, cortisol, and testosterone were measured). At that moment, the PRL value was 22.1 ng/mL. Regarding biochemical determinations, mild hyponatremia (Na 131 mEq/L) was noted. At this time, corticosteroid therapy was started at stress doses (hydrocortisone at 100 mg every 8 h intravenously). The patient underwent transsphenoidal surgery, and subtotal removal was achieved with glandular remnants on the bottom of the sellar region extended to the right cavernous sinus. The postoperative histological examination showed immunohistochemical expression of CAM 5.2, PRL, and ACTH.

The days after the surgery, mild hyponatremia was still present, with levels around 131–133 mEq/L, with no neurological symptoms associated. The level of PRL five days after surgery was 9.5 ng/mL. The thyroid and gonadotropic axes were also evaluated in this determination, showing a hormonal deficit of both axes at the central level. The patient was discharged with diagnoses of non-secretory pituitary macroadenoma with data of pituitary apoplexy, panhypopituitarism, and mild asymptomatic hyponatremia compatible with excessive fluid intake. Home treatment was hydrocortisone and levothyroxine. 

For the next year, PRL was measured several times, obtaining normal or minimally elevated values repeatedly: 15.8 ng/mL (August 2018), 27.6 ng/mL (January 2019), 71.2 ng/mL (July 2019). Dilution of the serum of the patient was performed to avoid the “hook effect”.

**Figure 2:**
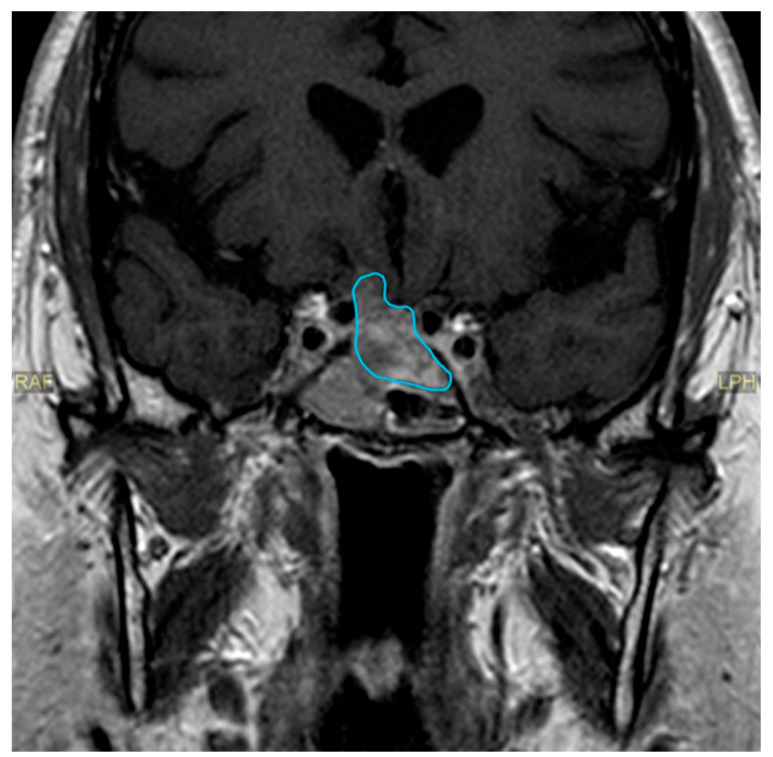
MRI, June 2020. Adenomatous recurrence with right optoquiasthmatic compressive effect, MRI, magnetic resonance imaging.

Almost two years after the surgical procedure, the patient reported that he had noticed a progressive visual deficit in his right eye for three weeks. A perimetry test was performed revealing a nasal defect in the right eye. A new MRI scan was requested (June 2020) (Figure 2), showing adenomatous recurrence with right optoquiasthmatic compressive effect, so the patient underwent a new transsphenoidal surgery. 

A probable right suprasellar adenomatous rest was evidenced in the post-surgery CT scan. This time, the postoperative histological examination showed fragments of extensively fibrous connective tissue without evidence of viable tumor cellularity, as well as respiratory submucosal glands without histological alterations. 

At that time, the patient was sent to our pituitary unit, and PRL was measured again after the new procedure, showing a relevant increase in the PRL level (565.3 ng/mL). The laboratory test was repeated, this time obtaining a PRL level of 681.9 ng/mL. A dynamic PRL release test was also performed, and the results were consistent with the isolated determination (PRL: 791.8 ng; PRL 30′: 702.4 ng/mL; PRL 60′: 667 ng/mL).

## 3. Results

### 3.1. Final Diagnosis

According to laboratory studies and imaging tests, the final diagnosis was a macroprolactinoma.

### 3.2. Treatment

At the time, treatment with a dopaminergic agonist (cabergoline) was started (October 2020). 

### 3.3. Outcome and Follow-Up

Only one month later, PRL levels were markedly decreased (0.4 ng/mL) (Table 1) and in the following perimetry test, a clear improvement in the visual deficit was shown (visual field index of 98% in the left eye and visual field index of 92% in the right eye).

**Figure 3:**
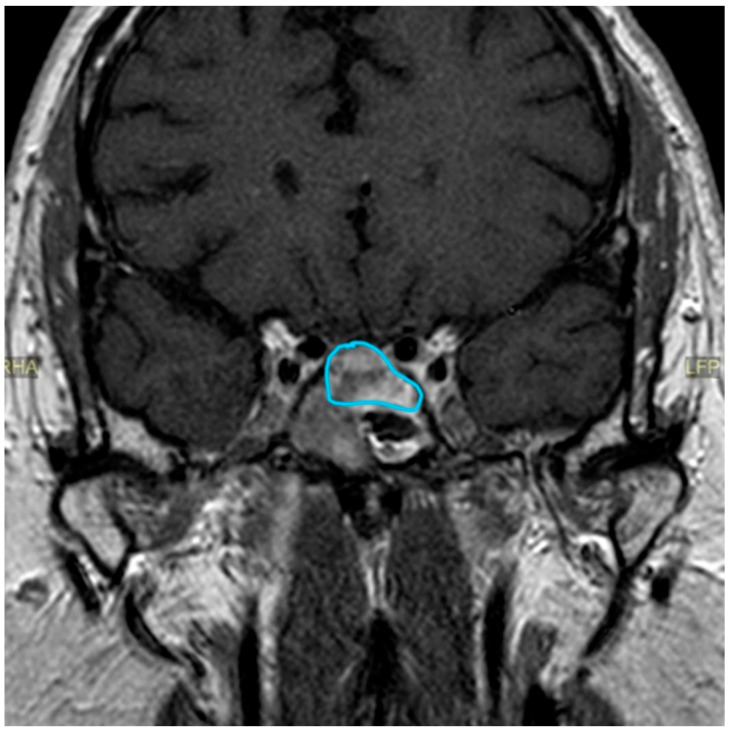
MRI, June 2021. The adenomatous rest reduced its volume, particularly in its cranial part, so it was no longer in contact with the gyrus rectus and the overlying subcallosal area. MRI, magnetic resonance imaging.

On the other hand, in terms of tumor size reduction, the benefit was more subtle: the adenomatous rest moderately reduced its volume, particularly in its cranial part, so it was no longer in contact with the gyrus rectus and the overlying subcallosal area (Figure 3).

The patient is currently on cabergoline treatment at a dose of 0.5 mg twice a week, with a good clinical evolution, maintaining a normal prolactin level as well as normal visual acuity.

## 4. Discussion

We present a not previously described clinical case of a pituitary macroadenoma with initial repeatedly normal PRL levels that during clinical evolution changed to markedly increased circulating PRL levels, compatible with macroprolactinoma with a good clinical response to dopaminergic agonists.

In the presence of a pituitary macroadenoma, markedly increased PRL makes the diagnosis of prolactinoma [10,11]. In men, prolactinomas are usually large and invasive, with signs and symptoms of hypogonadism and mass effects being the most frequent clinical features [12]. Additionally, according to the last World Health Organization classification [13], lactotroph tumors in men have a high probability of recurrence. In the diagnostic workup for prolactinoma, other causes of hyperprolactinemia should be excluded. A number of physiologic (stress, exercise, pregnancy, and breastfeeding) and pathologic conditions (cirrhosis, chronic kidney failure, primary hypothyroidism, compression of the pituitary stalk by a non-PRL-secreting pituitary tumor or different parasellar mass, and infiltration of the hypothalamus), as well as several drugs (antipsychotics, antidepressants, dopamine receptor blockers, dopamine synthesis inhibitors, and oral contraceptives) can induce symptomatic PRL level increase [9]. Attention should be paid to medical history, concomitant medications, and biochemical assessment [9]. In our patient, on initial clinical presentation, the PRL values were repeatedly normal, and the minimally elevated PRL values found on one occasion could have been due to compression of the pituitary stalk. 

Diagnostic workup for prolactinomas may be complicated by several challenges. Assay errors, macroprolactinemia, and high-dose “hook effect” are all possible reasons for false-positive or false-negative PRL measurements [14]. The “hook effect” is a possible explanation for the presence of artifactually low values of prolactin in the presence of a pituitary macroadenoma. Caution should be exercised in interpreting slightly increased serum PRL concentrations in the presence of a macroadenoma because of possible artifactually low values due to the “hook effect” [15,16,17]. This effect occurs when a very high PRL value, for example 4000 ng/mL or more, saturates both the capture and signal antibodies used in immunoradiometric and chemiluminescent assays, preventing the binding of the two antibodies in a “sandwich.” The result is an apparent PRL value that is only slightly increased, suggesting that the macroadenoma is an NFPA. The artifact can be avoided by repeating the analytical determination by performing dilution of serum [9]. In the present case, we performed dilution of the serum of the studied patient to avoid this “hook effect”.

NFPAs that completely changed their phenotype have been reported. Fang et al. [18] reported a patient with an initial NFPA. Several years later, the patient developed Cushing’s syndrome due to ACTH-dependent increased cortisol secretion, and the tumor extirpated during the fourth intervention showed marked immunopositivity for ACTH. To explain this event, some hypotheses have been formulated. The possibility that more than one tumor could be involved has been suggested in the differential diagnosis of patients with transformed-phenotype PAs [19]. Multiple or double adenoma are presented synchronously (although sometimes asynchronously). Most of them are clinically silent, small, and discovered incidentally [20]. Plurihormonal collision tumors from different lineages have been reported [21]; nevertheless, these pathological findings have not been described in cases reported as changing phenotype. Instead, multiple asynchronous PAs could be a plausible consideration in patients with transformed PAs. It is possible to assume a complex and multicausal mechanism. It has been proposed that a combination of gene alterations and several stimuli (drugs, pituitary surgery, or radiotherapy) may contribute to an accumulation of genetic changes, which may result in the functional variation observed in PAs [22]. Some PAs originate in an uncommitted stem cell, which can differentiate into two separate cell types. Also, one cell type can “trans-differentiate” into another cell type as a result of subsequent mutations during tumor progression. Starting from this concept, Dessimoz et al. [23] suggested the possible “trans-differentiation” into another cell type as an explanation for phenotype change in some PAs [23].

Silent PAs are tumors with positive staining for pituitary hormones or their transcription factors in the absence of hormonal hypersecretion. Silent somatotroph adenomas and silent lactotroph adenomas are rare [13]. On the contrary, PRL-producing pituitary adenomas without elevated PRL were found in a study in 12.1% of pituitary adenomas, and most patients were resistant to dopamine agonist treatment [24]. NFPAs are not associated with hormone hypersecretion, but sometimes, they even present slightly elevated PRL values [25]. Increased pressure on the pituitary stalk is probably the main mechanism for moderately increased PRL in NFPAs, although other factors, like young age, female sex, and decreased thyroid function, could participate [25]. An important aspect to be considered is that clinical or subclinical hemorrhage or infarction could modify the tumor hormone secretion and induce the phenotype change [18]. This could be a possibility in PAs with changing phenotype from functioning to nonfunctioning. However, it seems unlikely to explain the reverse transformation or cases with transformed phenotype [26]. These aspects highlight the rarity of the present case. 

Pituitary apoplexy may be the presenting feature of NFPAs or prolactinomas [27]. Between 2% and 12% of patients with all types of adenoma experience apoplexy, and the diagnosis of pituitary tumor was unknown at the time of apoplexy in more than three out of four cases [28]. Pituitary hemorrhage in prolactinomas has an overall prevalence rate of 6.8% and is significantly higher in macroprolactinomas (20.3%) compared with microprolactinomas (3.1%). These data show that incidental hemorrhage in prolactinomas is not uncommon. The present clinical case presentation was consistent with a clear pituitary apoplexy.

In our view, the most plausible explanation for the present case is that the pituitary apoplexy with hemorrhage of the macroadenoma at its initial presentation, with necrosis of the tumor lactotroph cells, provoked the normalization of the circulating PRL levels, which were previously elevated. Subsequently, as tumor regrowth slowly developed, marked hyperprolactinemia appeared. Thus, according to this explanation, no tumor transformation occurred. However, from a clinical point of view, the management of pituitary adenomas with markedly increased prolactin (prolactinoma) and with normal prolactin (NFPA) is completely different. We cannot completely rule out the possibility of an NFPA that completely changed its pathological and clinical phenotype to a prolactinoma or the presence of double adenomas not detected on pathological examination, as previously discussed. 

This case report represents an exception to the paradigm that in the presence of a macroadenoma and normal PRL levels (avoiding the “hook effect”), a prolactinoma can be discarded. Additionally, this case report shows the need for ongoing surveillance for these tumor types and the need for clinical suspicion for a different hormonal type. Moreover, it stresses the importance of comprehensive, regular, and lifelong vigilance of patients with NFPAs and the close monitoring of serum PRL concentrations.

Another secondary aspect that could be considered in relation to the medical treatment of PAs with dopamine agonists is cabergoline treatment. which has been employed as treatment for NFPAs [29,30,31], the present case highlights the possibility that some of the good results of such treatment for NFPAs [29,30] could be due to the inclusion of macroprolactinoma with normal circulating prolactin levels in some of those studies.

## 5. Conclusions

This case represents an exception to the widely accepted paradigm that in the presence of a macroadenoma and a confirmed normal PRL level (after avoiding the “hook effect”), a prolactinoma can be discarded. Moreover, it stresses the importance of comprehensive, regular, and lifelong surveillance of patients with NFPAs and the close monitoring and control of serum PRL concentrations. 

## Figures and Tables

**Table 1:** Prolactin levels, radiological evolution, and clinical management.

Date	July 2018	January 2019	July 2019	June 2020	October 2020	December 2020	June 2021
Prolactin (ng/mL)	22.1	27.6	71.2		681.9	0.4	1.8
MRI	Macroadenoma (20 × 20 × 18 mm) with signs of pituitary apoplexy. Invasion of the optic chiasm, hypothalamus, and the left cavernous sinus.	5 × 2 mm nodular structure that could be related to remnant glandular tissue. Persistent left deviation of the pituitary stalk.		Tumor growth (16 × 14 × 11 mm). Significant compression of the optic nerve and the optic chiasm.		Discrete decrease in volume of the tumor (13 × 12 × 8 mm). No compression of the optic nerve and the chiasm.	Volume reduction (13 × 10 × 6 mm). The structure is no longer in contact with the gyrus rectus and the subcallosal area.
Treatment	First surgical procedure			Second surgical procedure	Cabergoline started		

## Data Availability

The data used during the current study are available from the corresponding author upon a reasonable request.
